# Summer Diarrhœa

**Published:** 1902-08-23

**Authors:** 


### HOSPITAL CLINICS AND MEDICAL PROGRESS.

### SUMMER DIARRHŒA.

ALTHOUGH summer diarrhœa, which in some towns causes a very heavy mortality every year, is doubtless caused by some microbial poison, we do not as yet know the nature of the microbe engaged in its production, nor indeed do we know for certain whether the toxin is produced within the body of the patient or outside. In considering this disease, then, we may well confine our attention to its clinical phenomena and its treatment. Dr. Bosanquet[1] says that mild cases cannot be distinguished with any certainty from diarrhœa due merely to indigestible food, since even in the latter there may be some rise of temperature as well as looseness of the bowels. In mild cases the attack often begins with slight vomiting, the infant bringing up its milk in a curdled condition. The motions are unduly frequent, and different from the normal type, being often of a greenish colour and containing curds of undigested milk. There may be some colic, causing screaming, and drawing up of the legs; the temperature may be somewhat raised (100° to 102° Fahr.); the tongue is furred; the motions have a sour and sometimes an offensive smell, and traces of blood and mucous may be seen in them now and again, while if the case lasts more than two or three days they may become watery; there is weakness, restlessness, and irritability; the muscles feel flabby, the face is pale, and the pulse somewhat accelerated. There is thirst, and water may be retained even though milk may be at once vomited. Generally the illness yields to treatment, the temperature falls rapidly, and the attack is over in a couple of days. Even the mildest case may, however, at any time develop the most alarming symptoms, and be converted into the most virulent choleraic form within the space of a few hours.

In the severe cases, instead of rapid improvement taking place, all the phenomena become accentuated; the temperature remains high, 102° to 104° F. being not at all unusual. Sometimes, however, a subnormal temperature may be met with throughout. Vomiting continues, the motions remain frequent, becoming looser and more watery till they resemble dirty water with brownish particles suspended in it, or they may be frothy; they are very offensive, sometimes they contain mucus and blood in considerable quantities. The child becomes very feeble and lies in a drowsy condition, whining feebly if disturbed, but generally taking no notice of its surroundings. The cheeks, flushed at first, become pale and hollow, the eyes seem to sink into their sockets and are dull and lustreless, often kept continually half open, and towards the end the corneæ may be quite insensitive. The mouth is dry and coated with bands of fur, the fontanelle is sunk, and the cerebral pulsations are scarcely perceptible. The abdomen is generally swollen and tender, rarely it may be retracted. The urine is scanty and albuminous, and convulsions often usher in a fatal termination. The comatose state into which infants may fall towards the end of such attacks often much resembles the condition produced by meningitis, and it has been described as "hydrocephaloid" or "false hydrocephalus." It must not be supposed, says Dr. Bosanquet, that cases of this severe form are necessarily fatal. Recovery may take place even after the appearance of the gravest symptoms, though probably not when the hydrocephaloid state has become fully developed. The issue is generally determined one way or the other within a week or ten days. The diarrhœa may, however, last longer, and in some cases becomes chronic. A fatal result sometimes ensues from some complication, especially broncho-pneumonia. Otitis media is another possible complication.

The very acute cases, which have been described as "fulminating" or "choleraic," and to which some authorities would limit the term summer diarrhœa, are characterised by their suddenness of onset, by the rapid appearance of grave toxic symptoms, and frequently by the occurrence of death within the space of a few hours. Healthy and vigorous children seem as likely to suffer as those who are already enfeebled, and the wasting in such cases is very rapid, a fat, healthy baby being reduced within a few hours to a shrivelled wreck, scarcely recognisable by its parents. In regard to treatment Dr. Bosanquet says that the character of the motions serves to some extent to indicate the regimen. So long as they remain fæcal in character, modification rather than interdiction of milk may be practised, along with the use of drugs, among which lime-water may be included. But if the stools become watery and contain little fæcal matter it is advisable to suspend milk for 24 to 48 hours, giving in its place barley water or albumen water made by dissolving the white of one egg in a pint of water that has been boiled and then cooled. The solution is to be strained and may be flavoured. In severe cases with continued fever it may be necessary to withhold milk for several days, and it is surprising how well a diet of mere albumen water or barley water is tolerated. Cold water may be allowed for the relief of thirst.

In the early stages when some purgative is needed no drug is equal to calomel, say a sixth of a grain every hour or a quarter of a grain every three or four hours, according to the severity of the case. Opium should not be used in the early stages since it tends to diminish peristalsis and causes retention of intestinal contents. In later stages it is indicated in cases in which blood and mucus continue to appear in the motions, and also when the motions remain large and watery in spite of other methods of treatment. Opiate enemata may in such cases act better than administration of the drug by the mouth. Much reliance is now placed upon washing out the stomach and bowels with simple sterilised water, the stomach being dealt with by means of a soft catheter passed through the nose. For washing out the colon a small catheter and douche should be used, the catheter being passed as high as possible with great gentleness, and the fluid being allowed to flow out by its side when the gut is sufficiently distended. Several pints may thus be used. Sterilised normal saline is the best fluid (one drachm of salt to a pint of water). If there is much fever tepid sponging may be employed, or cool, but certainly not cold, fluids may be used for lavage of the colon. A wet pack may in some cases be desirable, the patient being kept in it for a considerable time, even several hours, and a small dose of stimulant being given at the same time. Stimulants are often required, and in some cases a quarter of a drop of liquor strychninæ ($=\frac{1}{400}$ of a grain of strychnine) may be usefully given hypodermically.

In a very practical article on the same subject Dr. Louis Fischer,[2] of New York, while agreeing that one of the first rules in the treatment of summer diarrhœa is to "stop milk feeding," points out that in acting thus we invariably do so at the expense of body weight. He therefore enters fully into the question of milk substitutes. Among those which he mentions are :—

*Barley Water.*—One heaping tablespoonful of ground barley flour to a pint of water. Boil for half an hour and strain through cheese-cloth. Sweeten with granulated sugar or with glycerine.

*Oatmeal Water.*—Prepared as above. The white of a raw egg—and even the yolk—may be well beaten up with either of the above.

*Almond Milk.*—Scald one or two ounces of sweet almonds ; remove the skins ; then add about eight ounces of fresh water and mash the almonds to a pulp ; boil this pulp with the water for fifteen or twenty minutes, strain through cheese cloth and add enough water to make one pint. Finally add one tablespoonsful of granulated sugar. This is a very nutritious proteid-containing substitute for milk.

*Meat Soups.*—A valuable substitute is good nutritious beef, mutton or chicken broth, which may if necessary be thickened with some farinaceous material, or perhaps better still may be boiled with mashed peas and strained.

*Tea and Coffee.*—Weak tea, with white of egg, or with dextrinised wheat, barley or rice, or with barley water. Coffee diluted with barley or rice water is very valuable when there is marked cardiac depression.

Among simple tests to be applied to milk Dr. Fischer advises the use of litmus paper. If this is turned red the milk is quite unfit for infant feeding. When milk has once turned no amount of boiling or pasteurising—however completely the bacteria may be killed thereby—can destroy the toxins which have been formed by the colon bacillus.

[1] Practitioner, August. [2] Med. Record, Aug. 2.

